# Detection of *cis*- and *trans*-acting Factors in DNA Structure-Induced Genetic Instability Using *In silico* and Cellular Approaches

**DOI:** 10.3389/fgene.2016.00135

**Published:** 2016-08-02

**Authors:** Guliang Wang, Junhua Zhao, Karen M. Vasquez

**Affiliations:** Division of Pharmacology and Toxicology, College of Pharmacy, The University of Texas at Austin, Dell Pediatric Research InstituteAustin, TX, USA

**Keywords:** non-B DNA, triplex, genetic instability, replication, DNA repair

## Abstract

Sequences that can adopt alternative DNA structures (i.e., non-B DNA) are very abundant in mammalian genomes, and recent studies have revealed many important biological functions of non-B DNA structures in chromatin remodeling, DNA replication, transcription, and genetic instability. Here, we provide results from an *in silico* web-based search engine coupled with cell-based experiments to characterize the roles of non-B DNA conformations in genetic instability in eukaryotes. The purpose of this article is to illustrate strategies that can be used to identify and interrogate the biological roles of non-B DNA structures, particularly on genetic instability. We have included unpublished data using a short H-DNA-forming sequence from the human *c-MYC* promoter region as an example, and identified two different mechanisms of H-DNA-induced genetic instability in yeast and mammalian cells: a DNA replication-related model of mutagenesis; and a replication-independent cleavage model. Further, we identified candidate proteins involved in H-DNA-induced genetic instability by using a yeast genetic screen. A combination of *in silico* and cellular methods, as described here, should provide further insight into the contributions of non-B DNA structures in biological functions, genetic evolution, and disease development.

## Introduction

Our understanding of the structure and function of genomic DNA has been dramatically advanced in the past few decades. More than 50% of genomic DNA contains repetitive sequences that do not necessarily code for protein (Lander et al., 2001), and were previously regarded as “junk DNA” (Lewin, 1986; Veitia and Bottani, 2009). In the past several decades, much research has supported the notion that these non-coding repetitive sequences play important biological roles in cells. In addition to the regulatory functions of non-coding RNAs (ncRNAs) such as microRNAs (miRNAs) that are transcribed from these “junk DNA” sequences (Perera and Ray, 2012; Hausser and Zavolan, 2014), many repetitive sequences are involved in chromosome architecture and organization and gene expression regulation due to their ability to adopt DNA conformations that differ from the canonical B-form DNA (i.e., non-B DNA).

### Non-B DNA Structure Formation at Repetitive Regions in the Genome

There are many published reports on the functions of non-B DNA-forming sequences in the regulation of gene expression, DNA replication, recombination, and telomere maintenance (van Holde and Zlatanova, 1994; Parkinson et al., 2002; Hanawalt and Spivak, 2008; Jain et al., 2008; Wang and Vasquez, 2009; Bochman et al., 2012; Boyer et al., 2013; Bansal et al., 2014). More than 15 types of alternative non-B DNA structures have been described to date (Choi and Majima, 2011; Wang and Vasquez, 2014); for example, simple repeats can form small “loop-out” bubbles caused by the misalignment of the repeat units (Mirkin, 2007), trinucleotide repeats can form imperfect hairpin structures, and inverted repeat sequences have the potential to adopt perfect self-complementary hairpins and cruciforms (Sinden et al., 1991; Voineagu et al., 2008; Lu et al., 2015). A Z-DNA structure with a left-handed zigzag-shaped backbone can form at alternating purine-pyrimidine regions such as GC or GT repeats, with the latter repeat representing the most abundant dinucleotide repeat in the human genome (Rich and Zhang, 2003). Z-DNA structures have also been demonstrated to form on CCG/CGG repeats in the presence of aluminum ions (Latha et al., 2002). Intramolecular triplex structures (H-DNA) can form at mirror-repeat homopurine/homopyrimidine sequences *via* formation of Hoogsteen hydrogen bonding of the purine-rich strand through the major groove of the underlying duplex (Voloshin et al., 1988; Frank-Kamenetskii and Mirkin, 1995). Because four guanine bases can associate through Hoogsteen-hydrogen bonding to form a square planar structure (guanine tetrad), four guanine-rich regions (from the same DNA molecule or different molecules) that contain runs of three or more guanines can stack to form G-quadruplex or G4 DNA structures (Lane et al., 2008; Bochman et al., 2012).

### Identification of Genomic *cis* Elements with the Potential to Adopt Non-B DNA Structures

The formation of non-B DNA structures requires appropriate repetitive sequence elements (as mentioned above), which allows the development of computational algorithms to search for genomic segments that have the potential to adopt non-B DNA structures. Many such *in silico* search algorithms are available on-line and can be used as a first step to uncover the biological functions of non-B DNA. For example, “einverted”^1^ or “palindrome”^2^ can be used to identify potential hairpin or cruciform-forming inverted repeats; “QGRS Mapper” searches for potential quadruplex-forming sequences^3^ (Kikin et al., 2006); and Cer et al. (2013) and our group have published algorithms to search for potential H-DNA-forming and Z-DNA-forming sequences^4^ (Wang et al., 2013) and Non-B DB v2.0^5^ (Cer et al., 2013).

### Non-B DNA and Genetic Instability Hotspots

An important discovery in the field of DNA structure is that many types of non-B DNA structures can lead to genetic instability in prokaryotic and eukaryotic cells in the absence of exogenous DNA damage (Wells et al., 2005; Wang and Vasquez, 2006; Zhao et al., 2010; Du et al., 2014; Lu et al., 2015). Genetic instability, from point mutations that change single base pairs to massive chromosomal aberrations, is a hallmark of many human diseases, including cancer. Thus, great effort has been expended to elucidate the mechanisms involved in DNA structure-induced genetic instability. Most models of genetic instabilities assume that DNA damage and mutation occur randomly, and those that confer survival/growth advantages are selected for, allowing continuous adaptation of tumor cells from normal tissue (Caporale, 2003; Wreesmann and Singh, 2005; Venkatesan et al., 2006). However, accumulating evidence provided by DNA sequencing of human cancer genomes (Sinclair et al., 2011) indicates that mutations are not distributed randomly in genomes. Common disease-associated “hotspots” where non-random mutations cluster have been reported in human genomes (Cleary and Sklar, 1985; Montoto et al., 2003; Popescu, 2003; Mefford and Eichler, 2009). Inspection of the sequences at or near those genetic instability hotspots has revealed that many non-B DNA-forming sequences are enriched at these regions, suggesting a correlation between non-B DNA structure-forming sequences and disease-associated genetic instability (Bacolla and Wells, 2004; Bacolla et al., 2004; Choi and Majima, 2011; Chen et al., 2013; Kamat et al., 2016). Computational analysis of human cancer DNA sequencing databases coupled with non-B DNA search algorithms can further reveal the connection between *cis*-acting non-B DNA-forming elements and genetic instability in human disease. Using this strategy, we have recently found that non-B DNA-forming sequences were significantly enriched within ±200 bp of breakpoints characterized in 19,947 translocations and 46,365 deletions in human cancer genomes, such as simple AT repeats that can form slippage structures or cruciform/hairpin structures, and GAA or GAAA repeats that can form slippage structures or H-DNA structures, and non-simple repetitive sequences that form H-DNA and Z-DNA conformations (Bacolla et al., 2016). We have also reported that short cruciform-forming inverted repeats are significantly enriched at translocation breakpoint hotspots in cancer genomes by searching within ±100 bp surrounding the translocation hotspots in ~20,000 human cancer genomes (Lu et al., 2015). Together, with findings that these non-B DNA structures are mutagenic in mammalian cells (Wang and Vasquez, 2004; Wang et al., 2006; Lu et al., 2015), these results clearly suggest that non-B DNA-forming sequences represent an intrinsic risk factor for genomic instability hotspots in human diseases.

### Non-B DNA-Forming Sequences Co-localize with Genetic Instability “Hotspots” in Human Cancer

**FIGURE 1:**
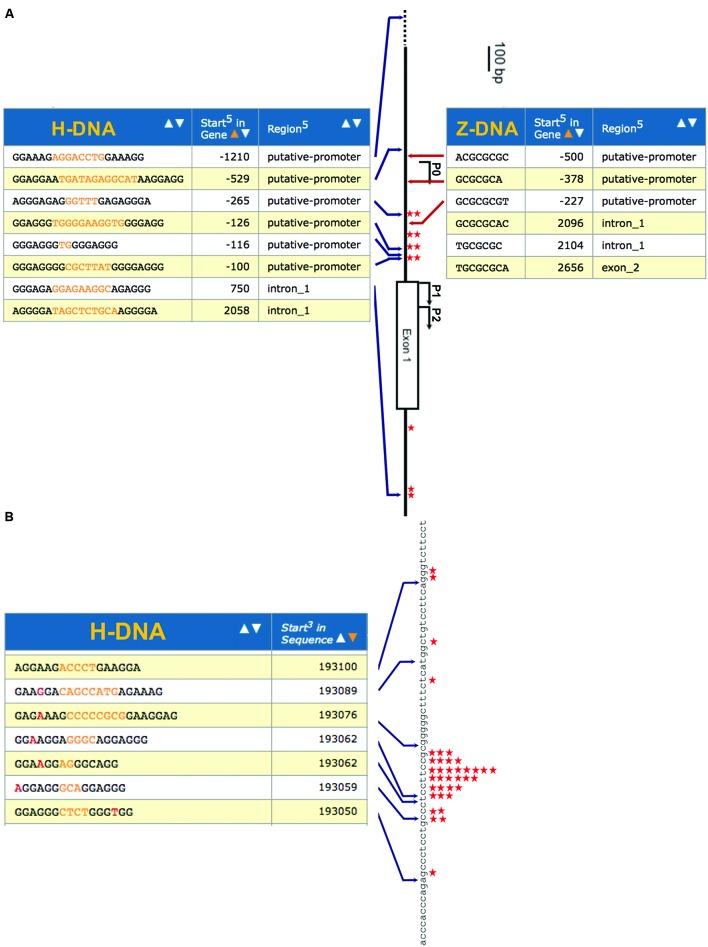
**Enrichment of non-B DNA structure at human mutation hotspots. (A)** Co-localization of H-DNA- and Z-DNA-forming sequences with mutation hotspots in the human *c-MYC* promoter. Search results for H-DNA- or Z-DNA-forming sequences in the promoter region of the human *c-MYC* gene are shown. P0, P1 and P2, Exon 1, and Intron 1 of the human *c-MYC* gene are shown in the center. Potential H-DNA-forming sequences are shown on the left and potential Z-DNA-forming sequences are shown on the right. Chromosome breakpoints involved in *c-MYC* translocations in previous reports, or single-strand-specific S1 nuclease sensitive sites in cultured human cells are marked as red stars. **(B)** Co-localization of H-DNA-forming sequences within the human *BCL-2* major breakpoint region. Search results for H-DNA-forming sequences in the upstream 70-bp from major breakpoint region of the human *BCL-2* gene are shown on the left. Chromosome breakpoints involved in *BCL-2* translocations in previous reports are marked as red stars on the right.

Here we used two oncogenes, *c-MYC* and *BCL-2*, as examples of co-localization of non-B DNA-forming sequences with genetic instability hotspots, because they are commonly involved in chromosomal translocations and the breakpoints in human cancer characterized. We analyzed a short 500-bp region from the human *c-MYC* promoter between P0 and P1 that represents a major breakage hotspot found in *c-MYC* translocation-induced lymphomas and leukemia (Care et al., 1986; Haluska et al., 1988; Joos et al., 1992; Saglio et al., 1993; Wilda et al., 2004). DNA double-strand breaks (DSBs) and subsequent translocation events can position the *c-MYC* gene under the control of a strong promoter of an immunoglobulin gene, leading to activation and overexpression of the oncogene in certain cancers (Care et al., 1986; Ramiro et al., 2006). H-DNA (Mirkin et al., 1987), Z-DNA (Rimokh et al., 1991; Wolfl et al., 1995) and G-quadruplex-forming sequences (Siddiqui-Jain et al., 2002) have been identified near this translocation breakage hotspot region. Using our non-B DNA search algorithm (Wang et al., 2013), we systematically inspected the *c-MYC* and *BCL-2* genes that are often involved in translocations in human cancers, for potential H-DNA- and Z-DNA-forming sequences (**Figure 1**). For H-DNA (shown on the left), we searched for homopurine/homopyrimidine sequences that contained mirror-repeat symmetries of a minimum repeat arm of 6 bp, and a 1–12 bp “spacer” sequence between the two mirror repeats. We allowed for one mismatch in symmetry when the “arms” were ≥10 bp, since a longer spacer between two mirror repeats or more mismatches could reduce the stability of an H-DNA structure. For Z-DNA (shown on the right), we searched for alternating purine-pyrimidine sequences that have a Z-DNA score of ≥75 [briefly, in contiguous alternating purine/pyrimidine fragments, each GC dinucleotide has a score of 25 and each GT or CA dinucleotide has a score of 3. For more details on the non-B DNA search algorithm and parameters for H-DNA and Z-DNA searches, please see (Wang et al., 2013)]. **Figure 1A** shows the search report of human *c-MYC* gene from the promoter to exon 1. Chromosome breakpoints involved in *c-MYC* translocations in human Burkett’s lymphoma and mouse plasmacytomas, or single-strand specific S1 nuclease sensitive sites in cultured human cells (Care et al., 1986; Haluska et al., 1988; Joos et al., 1992; Saglio et al., 1993; Michelotti et al., 1996; Kovalchuk et al., 1997; Wilda et al., 2004) are marked as red stars in **Figure 1A**. All previously reported H-DNA- forming (Mirkin et al., 1987) and Z-DNA-forming sequences (Wittig et al., 1992) identified by *in vitro* experiments were identified in the search, and were located on the *c-MYC* gene (**Figure 1A**). **Figure 1B** shows the search result of H-DNA forming sequences in the first 70 bps of the human *BCL-2* major breakpoint region (left), and the chromosome breakpoints within this area that are involved in *BCL-2* translocations (right, marked as red stars; Wyatt et al., 1992; DiCroce and Krontiris, 1995; Jager et al., 2000; Albinger-Hegyi et al., 2002; Raghavan et al., 2004). The co-localization of non-B DNA-forming sequences and chromosomal breakpoints identified in the *c-MYC* and *BCL-2* genes in translocation-related cancers (**Figure 1**), and a genome-wide significant enrichment of non-B DNA-forming sequences at genomic breakpoints in human cancer (Bacolla et al., 2016) suggest a role for non-B DNA in genetic instability and cancer development.

### Mechanisms of H-DNA-Induced Genetic Instability

DNA sequences that have the potential to adopt non-B DNA structures have been shown to impact DNA replication, transcription, DNA damage and DNA repair by recruiting and interacting with *trans*-acting proteins that bind, stabilize/destabilize or process non-B DNA conformations. Thus, identification of the proteins that interact with non-B DNA is critical to elucidate the mechanisms involved in non-B DNA-induced genetic instability. Because strategies to directly identify non-B DNA interacting proteins can be technically challenging, most approaches to date are based on functional analysis, i.e., by comparing the outcome of a non-B DNA structure-induced phenotype (mutagenesis) in cell lines/animals that are deficient or proficient in a gene of interest. Therefore, a fast, versatile and sensitive system to detect non-B DNA-induced genetic instability is warranted.

We have used a series of different mutation-reporter systems that can detect a variety of genetic instability events including base substitutions, point mutations, small and large deletions and insertions, and DSBs in bacteria, cultured mammalian cells, yeast and mice (Wang et al., 2009) and found that many different types on non-B DNA including H-DNA, Z-DNA and cruciform structures can stimulate genetic instability (Wang and Vasquez, 2004; Wang et al., 2006, 2008; Lu et al., 2015). Using these mutation-reporter systems, we have identified at least two different mechanisms of non-B DNA-induced mutagenesis: a DNA replication-dependent mechanism where the non-B DNA structure impacts the replication machinery, resulting in slippage and misalignment events at simple repeats, and replication fork stalling and collapse that can lead to DSBs; and a DNA structure-specific cleavage model that does not require DNA replication, in which the non-B DNA structures may be recognized as “damage” and cleaved by the DNA repair machinery (Wang and Vasquez, 2009, 2014; Vasquez and Wang, 2013). These models are discussed in more detail below.

### Replication-Related Mechanisms of H-DNA-Induced Genetic Instability

**FIGURE 2:**
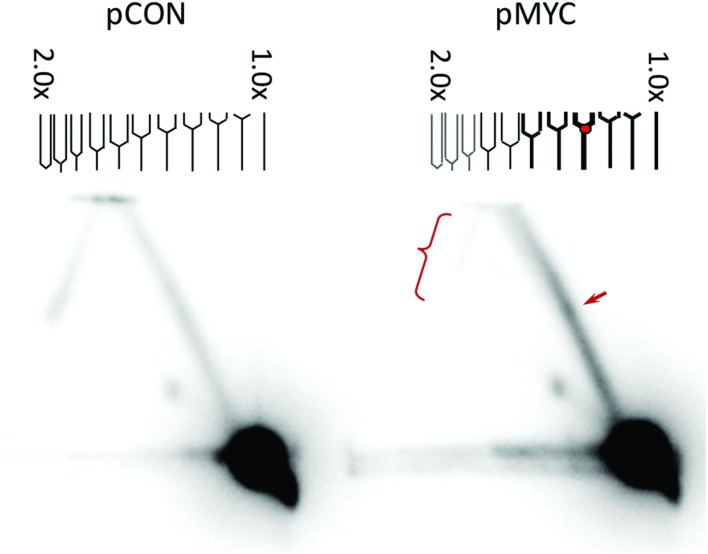
**H-DNA stalls DNA replication forks in mammalian cells.** Replication intermediates of pCON and pMYC plasmids recovered from mammalian COS-7 cells (24 h post-transfection) were separated via 2-D gel electrophoresis and the region containing the H-DNA-forming or control sequence was probed by Southern blotting. The SV40 Ori is not included in the probed regions, thus the products resulted in a typical Y-arc. The arrow designates the bulge on the Y-shaped replication arc, indicative of the accumulation of stalled replication intermediates. The lighter signal on the left arm of the arc (indicated with a bracket) on the pMYC sample suggests fewer replication forks passing through the H-DNA-containing region reducing the full-length products. A representative image of three independent repeats is shown.

To determine if H-DNA acts as an impediment to DNA replication, we measured replication fork stalling at a short H-DNA-forming sequence by using 2-D gel electrophoresis analysis and Southern blotting. The plasmid pMYC contains a short H-DNA-forming sequence from the human *c-MYC* promoter as identified in **Figure 1** (located at -100; Wang and Vasquez, 2004), while pCON contains a control B-DNA sequence. The plasmids were transfected into mammalian COS-7 cells and the replication intermediates of were recovered 24 h later, followed by *DpnI* digestion to remove unreplicated DNA. The *NdeI* and *BsaI* fragments containing the H-DNA or control inserts were separated by mass *via* gel electrophoresis in the first dimension, and by mass and shape by agarose gel electrophoresis in the second dimension, resulting in a typical Y-shape replication arc. As shown in **Figure 2**, H-DNA indeed stalled DNA replication, resulting in a bulge on the right arm of the arc and a much lighter left arm of the replication arc indicating stalling at/near the H-DNA insert resulting in fewer replication intermediates past the H-DNA sequence. In contrast, the control pCON plasmid formed a much smoother replication arc with near equal density of the left and right arms, indicating continuous replication. This result suggests that H-DNA can stall or pause the DNA replication machinery *in vivo*, perhaps in a fashion similar to our previous observation of H-DNA-induced RNA polymerase arrest within/near and downstream of the H-DNA-forming sequence (Belotserkovskii et al., 2007). These results are also consistent with our previous demonstration that this H-DNA-forming sequence can stimulate the formation of DSBs in an area ~100-bp up-stream and down-stream to the H-DNA structure (Wang and Vasquez, 2004). Thus, replication stalling-related DNA breakage is a likely mechanism for genetic instability induced by H-DNA in mammalian cells.

### H-DNA-Induced Genetic Instability Independent of DNA Replication

**FIGURE 3:**
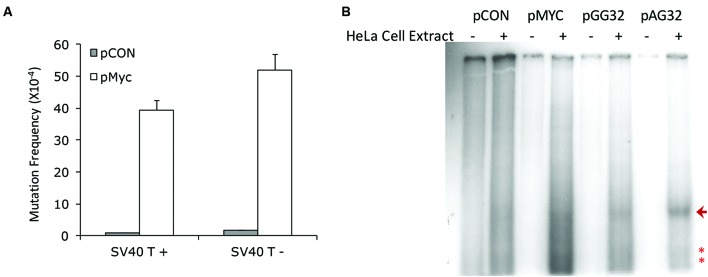
**Replication-independent H-DNA-induced genetic instability in HeLa cell extracts. (A)** H-DNA-induced mutation frequencies in the presence (SV40T+) or absence (SV40T-) of replication in HeLa cell-free extracts. The number of mutants observed and the total number of colonies screened are listed below the corresponding bars. Error bars show the standard errors of the mean value of three independent experiments. **(B)** H-DNA-induced cleavage in replication-incompetent HeLa cell extracts. A radiolabeled 400-bp fragment containing SSBs generated in HeLa cell extracts (+) is indicated by an arrow, and the radiolabeled shorter fragments indicative of H-DNA-induced DSBs in HeLa cell extracts are marked with “^∗^”. Plasmids incubated in the absence of HeLa cell extracts (-) served as controls.

Using same mutation-reporter shuttle vector as described above, we further investigated whether DNA replication was necessary for H-DNA-induced mutagenesis in mammalian cells. The SV40 replication origin on the vector allows control of the plasmid DNA replication, which for these studies provides an advantage over using endogenous genomic DNA elements. Here, we incubated the plasmids, pMYC and pCON, with HeLa cell free extract for 6 h. Plasmid DNA replication occurred only when the extract was supplemented with the SV40 T antigen. Interestingly, H-DNA stimulated mutations in both replication-competent and replication-incompetent extracts (**Figure 3A**). Although, direct comparison of the H-DNA-induced mutation frequencies in the replication-proficient and replication-deficient systems cannot be made (because the reporter plasmids recovered from the replicating system were digested with *DpnI* to remove the unreplicated DNA), this result indicated that H-DNA can stimulate genetic instability independent of DNA replication, similar to our results with Z-DNA- and cruciform-induced mutagenesis as we have previously reported (Wang et al., 2006; Lu et al., 2015).

To determine the spectrum of DNA breaks induced by H-DNA in the absence of replication, we radiolabeled the 5′-ends of the breakpoints on the plasmids incubated with the replication-incompetent HeLa cell extract and separated the ~400 bp *BsaI-BamHI* restriction fragments containing the H-DNA or control inserts by agarose gel electrophoresis. As shown in **Figure 3B**, all H-DNA-containing plasmids, pMYC, pGG32 and pAG32 plasmids (Wang and Vasquez, 2004) exhibited full-length restriction fragments and shorter fragments, indicating that both DNA SSBs and DSBs were generated near the H-DNA structure. These breakpoints were not seen in the plasmids that were incubated in the absence of cell extract, as expected. This result clearly suggested a replication-independent cleavage activity at/near the H-DNA structure that might be responsible, in part, for the mutagenesis observed in **Figure 3A**.

### Identification of *trans*-acting Factors Involved in H-DNA-Induced Mutagenesis

To identify the *cis*-acting factors involved in non-B DNA-induced genetic instability, we utilized a yeast artificial chromosome (YAC) to screen a yeast mutant library (Lu et al., 2015). The YAC-based reporter system provides a facile screening strategy in a eukaryotic system. It has advantages over the plasmid-based approach in that the non-B DNA sequence is integrated in a chromosomal context, and that knock-out yeast libraries are commercially available that allow complete depletion of the genes of interest (compared with an siRNA-depletion approach in human cells). The system we used here contains H-DNA (or a B-DNA control sequence) integrated between a *URA3*-reporter gene and a C4A4 telomere seed sequence to rebuild the telomere after loss of the distal arm in the YAC (Wang et al., 2009; Lu et al., 2015), which allows for detection of non-B DNA-induced DSBs (Wang et al., 2009; Lu et al., 2015). H-DNA-induced DSBs will result in the loss of *URA3*, which can be selected for by growth in medium containing 5-Fluoroorotic Acid (5-FOA). As shown in **Table 1**, in wild-type (WT) yeast BY4742 cells, a short H-DNA-forming sequence from the human *c-MYC* promoter induced ~9.4-fold more FOA-resistant *URA3-* colonies over that of the control, consistent with our findings from mutation-reporter plasmids in mammalian cells (Wang and Vasquez, 2004). The same YAC reporter constructs were then transformed into various mutant yeast strains deficient in genes of interest via a kar-cross method (Wang et al., 2009; Lu et al., 2015) and H-DNA-induced genetic instability was measured. Results from an initial screen are listed in **Table 1**.

**Table 1:** An H-DNA-forming sequence induces DNA instability in WT and deficient yeast.

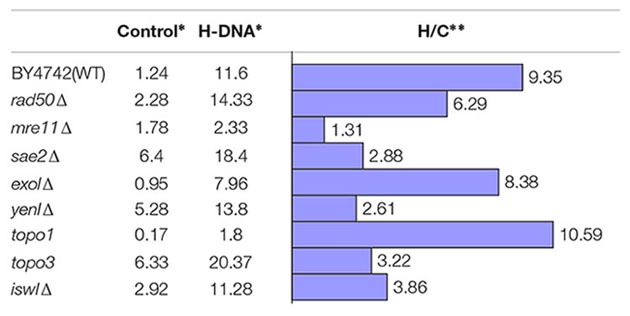

*^∗^Number of yeast colonies with loss of Ura3 in 100,000 cells. At least 30,000 colonies were screened in each fragility assay.*

*^∗∗^H/C represents the fold increase in genetic instability induced by H-DNA over control in the various yeast strains. H-DNA-induced significantly more mutants in all WT and deficient yeast strains. P-values < 0.05 in two-sided t-test comparing H-DNA vs. Control, from >3 repeats. H-DNA-induced mutation above control in all deficient yeast strains list above are significantly different from that from WT cells. P-values < 0.05 in Fisher’s exact test from >3 repeats*.

As we have reported previously, H-DNA can stimulate DSBs in mammalian cells, with the vast majority of mutations being large-scale deletions containing microhomologies at the junctions, suggesting a role for a micro-homology-mediated end-joining (MMEJ) mechanism in generating H-DNA-induced mutations *in vivo* (Wang and Vasquez, 2004). Thus, we tested H-DNA-induced genetic instability in yeast strains deficient in proteins involved in such pathways. We found that deletion of *mre11* diminished the H-DNA-induced mutagenesis to background levels (only 1.3-fold above the control sequence in *mre11Δ)*, suggesting that Mre11 is involved in the H-DNA-induced mutagenesis (**Table 1**). Yeast strains deficient in *rad50* and *sae2* also reduced the H-DNA-induced genetic instability, though not as dramatically as in the *mre11Δ* cells, suggesting roles for these proteins in H-DNA processing and H-DNA-induced mutations. The Mre11-Rad50 complex has been shown to cleave hairpin structures at the junction of the center loop and stem, and the duplex stem and the 3′ single-strand overhang (Trujillo and Sung, 2001), indicating a ssDNA-dsDNA junction-specific activity. Sae2 is an endonuclease that interacts with the Mre11-Rad50-Xrs2 (MRX) complex in processing hairpin DNA structures during DSB repair (Lobachev et al., 2002). Thus, it is possible that similar ssDNA-dsDNA junctions found in H-DNA could be recognized and cleaved by this protein complex, leading to genetic instability. Similarly, *exo1* that encodes a 5′–3′ exonuclease and shares functional overlap with Mre11 and Rad27 nucleases in DNA metabolism (Moreau et al., 2001), might also be involved H-DNA-induced mutations, as indicated by the reduced level of H-DNA-induced genetic instability in the *exo1Δ* cells (**Table 1**).

We utilized the YAC screen in yeast deficient in enzymes that are involved in other DNA processes, for example, *Yen1* Holliday junction resolvase, which is the homolog of human *GEN1*. Overexpression of GEN1 was reported to resolve the accumulated cruciform-shaped replication intermediates at damaged forks caused by *Sgs1*-deficiency in yeast cells (Mankouri et al., 2011). We found that deficiency of *yen1* reduced the level of H-DNA-induced mutagenesis in our YAC-reporter system, suggesting a potential role in resolving H-DNA structures. However, further work is warranted to determine if Holliday junction resolvases can cleave the branched structure on H-DNA and contribute to genetic instability.

Topoisomerases can relax the negative supercoiling on duplex DNA (Wang, 2002), which might assist in resolving non-B DNA conformations, including H-DNA. Consistent with this notion, we found that *topo1* deficiency, one of the major nuclear enzymes that relieves torsional strain in DNA, led to a modest increase in H-DNA-induced genetic instability. Since topoisomerases have been shown to cleave non-B DNA structures (Pognan and Paoletti, 1992; Froelich-Ammon et al., 1994), genetic instability could result if the breakpoints were not faithfully rejoined. Here we found that deficiency of *topo3* that codes for a conserved protein (in a complex with Sgs1 and Rmi1) that is primarily implicated in homologous recombination and disassembly of branched DNA structures (Mankouri and Hickson, 2007; De Muyt et al., 2012) also contributed to H-DNA-induced genetic instability (**Table 1**).

The formation of non-B DNA structures is a dynamic process and is affected by many factors including chromatin-remodeling proteins. The *ISW1* gene codes for an ATPase subunit of the largest class of mammalian remodelers ISWI (Goldman et al., 2010) and is involved in transcription elongation (Morillon et al., 2003; Smolle et al., 2012). We found that the absence of *isw1* substantially reduced the H-DNA-induced genetic instability compared to WT yeast cells. Future studies are warranted to determine the extent to which the direct chromatin remodeling function and/or the transcription elongation function of *ISW1* led to this effect.

The field of DNA structure-induced genetic instability is still at an early stage and much more work is required to determine the mechanisms involved for each type of non-B DNA structure. The gene products identified in the YAC screening system still need to be confirmed in other systems (e.g., mammalian cell systems or mouse models), and the mechanisms of how these proteins are involved in H-DNA formation and processing need to be explored. Updated databases of mutation hotspots in human disease, coupled with information on DNA modifications, epigenetics, and chromosome structure, will allow for further advances to be made in understanding how non-B DNA-forming sequences impact disease etiology. New methods to detect non-B DNA conformations, particularly in living cells, will facilitate the elucidation of the biological functions of these non-B DNA structures.

## Materials and Methods

### *In vitro* Mutagenesis Assay in HeLa Cell-free Extracts

The mutation-reporter plasmids contain an SV40 origin of replication and can only be replicated in the presence of the SV40 large T antigen. 50 ng of plasmid DNA was incubated with 180 μg SV40 large T antigen-deficient HeLa cell-free extract from a CHIMERx DNA replication assay kit (Milwaukee, WI, USA) supplied with 1 μg SV40 large T antigen (for the replication-competent system) for 6 h at 37°C in a reaction buffer (30 mM HEPES, pH 7.5, 7 mM MgCl_2_, 0.5 mM dithiothreitol, 4 mM ATP, 100 μM each of dNTP, 50 μM each of NTP, 40 mM phosphocreatine, 0.625 units of creatine phosphokinase). Purified large T antigen (CHIMERx) was added to the manufacturer’s recommended reaction mixture to allow for DNA replication. H-DNA-induced mutants were determined by blue-white screening and analyzed by restriction digestion analysis and DNA sequencing as we have described (Wang and Vasquez, 2006).

### *In vitro* H-DNA-Induced Incision Assay in HeLa Cell Extracts

Fifty ng of plasmid DNA pCON, pMYC, pGG32 or pAG32 was incubated with 180 μg HeLa cell-free extract as in the replication assay (see above) for 4 h in the absence of SV40 large T antigen. After purification, the 5′-ends produced at the breakpoints in HeLa extract were radiolabeled with T4 polynucleotide kinase and [γ-^32^P] ATP and then the plasmids were digested with *BsaI* and *BamHI* to release an ~400-bp fragment containing the H-DNA or control insert. Products were separated on 1.5% agarose gels and subjected to autoradiography. Plasmids incubated under similar conditions but in the absence of HeLa cell extract were included as controls.

### Two-Dimensional Gel Electrophoresis Analysis of Replication Intermediates in Mammalian Cells

Plasmids were transfected into mammalian COS-7 cells using GenePORTER (GTS, Inc., San Diego, CA, USA) according to the manufacturer’s recommendations. After 24 h, replication intermediates were isolated and subjected to 2D gel electrophoresis according to Krasilnikova and Mirkin (2004). After being transferred to membranes, replication intermediates were identified by radiolabeled probes specific to this NdeI-BsaI fragment.

### Yeast Strains and YACs

Yeast strain 213 (MATa *Kar1-1 Leu2-3,112 Ura3-52 his7*), was used for investigation of the mutagenic capability of the H-DNA sequences and for kar-crossing with other yeast strains to introduce the reporter YACs. Yeast strain BY4742 (*MATα, his3Δ1, leu2Δ0, lys2Δ0, ura3Δ0*) and its derivatives were used to determine the mutation frequency variance between the WT strain and the mutant strains. YACs containing the H-DNA or control sequences were constructed by cloning these sequences between the C4A4 and *URA3* genes on a replication-defective pRS306 plasmid. *AatII* linearized plasmids were introduced into a *URA3-* host strain containing a YAC with a non-functional point-mutated *URA3* gene. Homologous recombination between YAC VS5 and the pRS306 plasmid generated a YAC containing the insert and a functional *URA3* gene. After transformation, URA3+ transformants were selected on SD+DO-Leu-Ura plates and the presence of H-DNA or the control B-DNA sequence on the YAC was confirmed by colony PCR followed by direct DNA sequencing, as previously described (Lu et al., 2015).

### YAC Instability Assay

Donor cells K213 (MATa *Kar1-1, his7, leu2-3, 112, ura3-52*) containing YACs with the H-DNA-forming sequence or the control B-DNA-forming sequence were grown from a single colony overnight at 30°C. Canavanine (Sigma) resistant recipient cells (BY4742 background, MATα *his3-Δ1, leu2-Δ2, ura3-Δ0*, ATCC) from the deletion library (GSA-5, ATCC, Manassas, VA, USA) were grown in YEPD at the permissive temperature overnight. YACs were transferred from donor cells to recipient cells via kar-crossing and were selected on SD+DO-Ura-Arg+canavanine (60 mg/L) plates. The YAC transfer was confirmed by survival on SD+DO-Ura-Leu plates, but not on SD+DO-Lys plates. Insertion sequences on the YACs were further confirmed by PCR amplification. Five (5) starting colonies were used to inoculate cultures that were grown overnight at the permissive temperature in SD+DO-Leu. 50 μl of each culture was plated on SD+DO-Leu with 5-fluoroorotic acid (5-FOA, Zymo Research, Irvine, CA, USA) plates to select for breakage events, while another 50 μl of each culture was combined, and the mixture was diluted and plated on SD+DO-Leu plates for a total cell number count. The mutation frequency of non-B DNA was calculated as the number of FOA resistant (FOA^R^) colonies divided by the number of total colonies.

## Author Contributions

GW, JZ, and KV designed the study; GW and JZ performed experiments; GW and KV analyzed data, discussed the results and wrote the manuscript.

## Conflict of Interest Statement

The authors declare that the research was conducted in the absence of any commercial or financial relationships that could be construed as a potential conflict of interest.

## Footnotes

1 http://emboss.bioinformatics.nl/cgi-bin/emboss/einverted

2 http://emboss.bioinformatics.nl/cgi-bin/emboss/palindrome

3 http://bioinformatics.ramapo.edu/QGRS/index.php

4 http://sites.utexas.edu/adrgs/research-tools/dna-structure-search/

5 http://nonb-abcc.ncifcrf.gov
